# Detection of high variability in gene expression from single-cell RNA-seq profiling

**DOI:** 10.1186/s12864-016-2897-6

**Published:** 2016-08-22

**Authors:** Hung-I Harry Chen, Yufang Jin, Yufei Huang, Yidong Chen

**Affiliations:** 1 Greehey Children`s Cancer Research Institute, The University of Texas Health Science Center at San Antonio, San Antonio, TX 78229 USA; 2 Department of Electrical and Computer Engineering, The University of Texas at San Antonio, San Antonio, TX 78249 USA; 3 Department of Epidemiology and Biostatistics, The University of Texas Health Science Center at San Antonio, San Antonio, TX 78229 USA

**Keywords:** Single-cell, Single-cell RNA-Seq, Cell heterogeneity, Negative binomial distribution, Gene expression variation model, Variably expressed genes

## Abstract

**Background:**

The advancement of the next-generation sequencing technology enables mapping gene expression at the single-cell level, capable of tracking cell heterogeneity and determination of cell subpopulations using single-cell RNA sequencing (scRNA-seq). Unlike the objectives of conventional RNA-seq where differential expression analysis is the integral component, the most important goal of scRNA-seq is to identify highly variable genes across a population of cells, to account for the discrete nature of single-cell gene expression and uniqueness of sequencing library preparation protocol for single-cell sequencing. However, there is lack of generic expression variation model for different scRNA-seq data sets. Hence, the objective of this study is to develop a gene expression variation model (GEVM), utilizing the relationship between coefficient of variation (CV) and average expression level to address the over-dispersion of single-cell data, and its corresponding statistical significance to quantify the variably expressed genes (VEGs).

**Results:**

We have built a simulation framework that generated scRNA-seq data with different number of cells, model parameters, and variation levels. We implemented our GEVM and demonstrated the robustness by using a set of simulated scRNA-seq data under different conditions. We evaluated the regression robustness using root-mean-square error (RMSE) and assessed the parameter estimation process by varying initial model parameters that deviated from homogeneous cell population. We also applied the GEVM on real scRNA-seq data to test the performance under distinct cases.

**Conclusions:**

In this paper, we proposed a gene expression variation model that can be used to determine significant variably expressed genes. Applying the model to the simulated single-cell data, we observed robust parameter estimation under different conditions with minimal root mean square errors. We also examined the model on two distinct scRNA-seq data sets using different single-cell protocols and determined the VEGs. Obtaining VEGs allowed us to observe possible subpopulations, providing further evidences of cell heterogeneity. With the GEVM, we can easily find out significant variably expressed genes in different scRNA-seq data sets.

## Background

Single-cell analysis has emerged a decade ago to understand the heterogeneity of a cell population, especially in biology contexts such as early embryonic development and tumor etiology [1]. Single-cell quantitative PCR (qPCR) [2–4] or single-molecule RNA fluorescence in situ hybridization (FISH) [5] have been widely used as low-throughput approaches to measure the expression of specific genes at a single-cell level. Although experiments using these methods can provide crucial information of cellular heterogeneity and the presence of distinct cell subpopulations, only a small number of genes can be monitored simultaneously. RNA sequencing (RNA-seq), a developed approach using next-generation sequencing (NGS) technology, can unbiasedly detect the genome-wide gene expression of a sample. Bulk RNA-seq experiments start with a large population of cells (> 10^5^), and the gene expression levels are considered as the average expression across the population of a cell pool [6]. Bulk RNA-seq might be sufficient in many contexts such as revealing the aberration of mRNA expression between different treatments, conditions, or phenotypes. However, biological questions like diversity in early stage development embryonic cells, which each cell has distinct functions, can't be explained by bulk RNA-seq experiments. With recent introduction of Smart-seq protocol, the required volume of starting materials has been vastly reduced, making the single-cell RNA sequencing (scRNA-seq) achievable [7, 8].

There are already several protocols for sequencing of single cells, which allow researchers to assay high-throughput gene expression profiling at the single-cell level of a large number of cells. However, unlike the conventional RNA-seq where analysis tools are abundantly available, the lack of bioinformatics tools for single-cell RNA-seq limits its huge potential. Comparing with bulk RNA-seq measurements, single-cell RNA-seq data tend to have much lower read counts (~200,000 to 5 million reads per cell) [9], higher variability, and large number of outliers, and all these are poorly accommodated by conventional RNA-seq analysis methods [10]. Unlike the objectives of conventional RNA-seq where differential expression analysis and the detection of differentially expressed genes (DEGs) are integral components, the most important goal of scRNA-seq is to identify variably expressed genes (VEGs) across a population of cells to account for the discrete nature of single-cell gene expression and uniqueness of sequencing library preparation protocol for single-cell sequencing. As we observed, the transcriptional heterogeneity of the cell population can be assessed by the expression variation difference, whether they are lowly or highly expressed, which conventional RNA-seq analysis failed to identify due to the assumption of homogeneity within each cell subtype.

In recent studies, gene expression variation models were proposed specifically for the corresponded scRNA-seq experiments in order to detect VEGs deviated from the Poisson model [11, 12]. However, different scRNA-seq data sets rendered different distributions and a common mathematical description is necessary. Hence, the purpose of this study is to provide a mathematical description of a gene expression variation model (GEVM) for scRNA-seq data. The model addresses the over-dispersion of single-cell data and the additional variability caused by different sources of variation. By exploiting existing statistical tools such as local regression and nonlinear least squares curve fitting, the parameters of gene variation model are estimated and statistical significant VEGs can be identified. To study the robustness of the model, we have built a simulation framework to generate single-cell RNA-seq data using different distributions in each step to imitate the dispersion of real data in different conditions. We demonstrated robustness of our method by applying it to the simulated data and test how precise we can estimate the parameters to the initial settings.

## Methods

### Modeling of single-cell RNA-seq data

To develop a generic GEVM, we exploited the over-dispersion concept from edgeR [13]. Assuming each gene's expression follows a negative binomial (NB) distribution with parameter NB(*r*_*i*_, *p*_*i*_) for *i*^th^ gene, we have

1 $$ {\sigma}_i^2=\frac{\mu_i}{1-{p}_i}={\mu}_i+\frac{\mu_i^2}{r_i}, $$

where the *μ* and *σ*^2^ are gene expression mean and variance, respectively. We further assume that in a given condition across a cell population, the model parameter *r* does not change (invariant to gene expression level), or

2 $$ {\sigma}^2=\mu +\alpha {\mu}^2, $$

where *α* is defined as the dispersion, or *α* = 1/*r*. For simplicity, we omitted gene index from Eq. 2. Clearly, when *α* > 0, the data are from a NB distribution. If *α* = 0, the data can be represented by a Poisson distribution (or *r* → ∞), which follow the diagonal line with a slope of $-\frac{1}{2}$ in a log-log CV-mean plot where *σ*^2^ = *μ* in Eq. 2. However, there are many sources of technical variation that contribute to the variability of scRNA-seq data. For instance, single-molecule capture efficiency, 3』 end bias due to single-cell RNA library preparation protocol, and low expression of genes that are easily affected by noises [14]. In this respect, we assume *σ*^2^ = *μ* + *bμ* = *βμ*, where *β* = 1 + *b*, and *bμ* is an additive noise component (proportional to the mean signal strength). Thus, the data deviate from the original diagonal line, following a line of ${ \log}_{10}(CV)=-\frac{1}{2}{ \log}_{10}\left(\mu \right)+\frac{1}{2}{ \log}_{10}\left(\beta \right)$. Consequently, we extended the relation between the mean and variance given in Eq. 2, by adding a model parameter *β* to represent the multiplicative effect of different sources of technical noises.

3 $$ {\sigma}^2=\beta \mu +\alpha {\mu}^2, $$

where we also assumed *β* is invariant within each cell population. We further obtained, from Eq. 3, the relationships between the coefficient of variation (CV, defined as *σ*/*μ*) of each gene across the cell population and its average expression level as follows,

4 $$ { \log}_{10}(CV)=\frac{1}{2}{ \log}_{10}\left(\frac{\beta }{\mu }+\alpha \right). $$

Therefore, by measuring the CV and mean abundance of gene expression *μ* from all genes, we can estimate the two parameters *α* and *β* and dissect the baseline of the cell population. Note that from Eq. 4 when the mean expression level *μ* becomes larger, $CV\to \sqrt{\alpha }$, or a constant coefficient variation [15], and when *μ* ≪ 1 the *σ*^2^ → *βμ*, or equivalently to a Poisson parameter *λ* ' = *βμ*.

### Estimation of model parameters and selection of significant VEGs

**Fig. 1:**
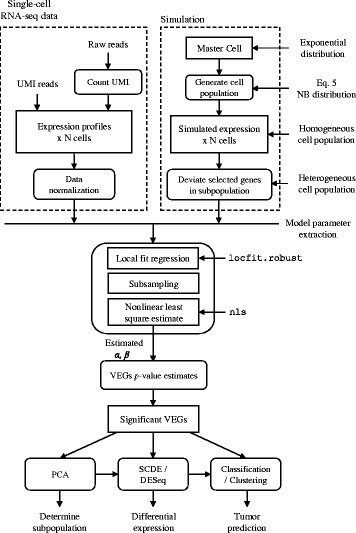
Workflow of identifying significantly variably expressed genes and the following analyses for single-cell RNA-seq data

In order to identify genes whose variation of gene expression are larger than those defined by Eq. 4, we need to estimate model parameter α and β from a scRNA-seq data set derived from a given cell population. The estimation procedure is as follows (Fig. 1): firstly we calculate the mean and coefficient of variation of each gene across a set of cells; afterwards, we perform a robust local regression implemented in locfit (R package) for fitting a robust CV-mean relationship. The nonlinear curve starts at the point with enough neighboring points (>0.5 % of total genes) to prevent overemphasizing the low expression section due to the subsampling in the next step. In addition, we also terminate the nonlinear curve at the smallest CV point to constrain to a flat line. As a typical phenomenon in scRNA-seq, only a few genes with high expression levels, results in an inaccurate local fitting at the right-tail side. On the other hand, a large proportion of genes locates in the middle section, leading to a bias during least-squares fitting in the next step. To remedy this bias, we subsample the fitted data points in a fixed interval (0.01 in log_10_ scale) from the start to the terminal point. Then we employ nonlinear least-squares fitting implemented in nls (R package) to estimate the two model parameters (*α* and *β)* of the GEVM. Now we can get the CV difference *D*_*i*_, which is the shortest distance of gene *i* to the ideal model with parameter *α* and *β* as a measure of variability.

### Determination of *p*-value of VEGs

Instead of picking VEGs by the rank of CV difference *D*_*i*_, we hypothesize that under the assumption of a homogeneous cell population, the CV difference to the model curve (Eq. 4) possesses a normal distribution (around baseline). We further assume that majority of genes, in a heterogeneous cell population, do not deviate much. Therefore, we use the CV differences of the data points around the model curve (Eq. 4) to fit a normal distribution. Even though robust local regression is used to estimate the expression variation model, the model is still influenced by those outlier genes. Hence, we use kernel density to find the center of the normal distribution. Afterwards, we fit the normal distribution using the CV differences below the center point. We can calculate *p*-value of each data point from the normal distribution and determine the significance of VEGs comparing to initial homogenous cell population. The procedure of Benjamini and Hochberg [16] is also applied to obtain the false discovery rate (FDR). Fig. 1 shows the overall workflow for detecting VEGs in scRNA-seq data set.

### Simulation of scRNA-seq data from a homogeneous cell population

In order to evaluate the robustness of our GEVM, we generated a set of simulated data where we could control the baseline parameters and the differential expression status for a set of genes in a random set of cells. First, we utilized exponential distribution (with 3 different mean values: 0.25, 1, and 10, respectively) to create a “master cell” and its genome-wide expression levels of a cell population. The two lower mean values were designed to reflect the nature of low expression events in scRNA-seq data. The master cell expression level *M*_*i*_ would be the base expression value of gene *i* for the other single cells in the population (children cells derived from a single master cell).

Given the master cell expression level *M*_*i*_, and the assigned parameters *α*, *β*, the children single cells *x*_*ij*_ were simulated with a negative binomial distribution,

5 $$ {x}_{ij} \sim NB\left({r}_{ij},\ {p}_{ij}\right) $$

where the two NB parameters *r*_*ij*_ and *p*_*ij*_ were further computed by,

6 $$ {r}_{ij}=\frac{\mu_{ij}^2}{\sigma_{ij}^2-{\mu}_{ij}}=\frac{\mu_{ij}}{\beta -1+\alpha {\mu}_{ij}} $$

7 $$ {p}_{ij}=\frac{\mu_{ij}}{\sigma_{ij}^2}=\frac{1}{\beta +\alpha {\mu}_{ij}} $$

Equations 6 and 7 were obtained utilizing our model Eq. 3. The mean value of gene *i* in cell *j*, *μ*_*ij*_, was derived from the master cell expression level with a Gaussian distribution of *μ*_*ij*_ = *N*(*M*_*i*_, *max*(0.2, 0.2 * *M*_*i*_)). Here we required standard deviation greater than 0.2 to avoid small or near 0 standard deviation.

### Simulation of scRNA-seq data from a heterogeneous cell population

To generate a cell population with non-distinct grouping effects, we first select a percentage of cells to be deviated from its original homogeneous population governed by the master cell. To achieve that and with a set of selected cells, we determine a subset of genes (variable *prct*) whose expression levels to be altered, and we generate the log fold change of each selected gene from a normal distribution to simulate a gradual fold change, with majority of them with minimal alteration. The fold change of a selected gene *k* is generated as,

8 $$ { \log}_2\left(F{C}_k\right) \sim Normal\left(\mathrm{mean}=0,\ s=2\right) $$

where the variation level can be controlled by modifying the standard deviation *s* of the normal distribution. To determine a subset of cells to be altered, the probability of each cell to be deviated is in a uniform distribution, *uniform*(0, 1) and a cell with probability larger than 0.9 is classified as a heterogeneous cell.

By using different distributions for simulation, we are able to generate data close to real scRNA-seq data under different conditions by changing the assigned parameters. We also compare our model with the noise model (Eq. 9) from a previous study [12]. At last, we measure the root mean square error (RMSE) to test the robustness of both methods on the simulated data, where RMSE is evaluated against log_10_(*CV*) over *μ* at a fixed interval, between input and estimated models.

9 $$ { \log}_{10}(CV)={ \log}_{10}\left({\mu}^{\gamma }+\delta \right) $$

### Single-cell RNA-seq data set for testing

Two mouse scRNA-seq data sets were obtained from Gene Expression Omnibus (GSE65525 and GSE60361) [11, 12]. GSE65525 is the mouse embryonic stem cells with 24,175 genes in 933 single cells, sequenced using CEL-seq protocol [17], and GSE60361 is the mouse cerebral cortex cells with 19,970 genes in 3,007 cells, sequenced using quantitative single-cell RNA-seq protocol [18]. Both data sets were counted using unique molecular identifiers (UMIs) to eliminate duplicated reads caused by library amplification. Following previous study [11], we also performed the same scaling normalization method on both UMI count data sets,

10 $$ {\widehat{k}}_{ij}={k}_{ij}\overline{K}/{K}_j,\kern0.1em \mathrm{where}\kern0.2em {K}_j={\displaystyle \sum_i}{k}_{ij} $$

where *k*_*ij*_ is the UMI count of gene *i* in cell *j*, *K*_*j*_ is the total UMI count of cell *j* and $\overline{K}$ is the average UMI count among the cell population. Genes that expressed in less than 1 % of the cell population were removed before applying to the model. As we shown later, the two data sets distribute differently. Under these two distinct cases, we will test the performance of the proposed method under different conditions.

## Results and discussion

### Implementation of noise model on simulation data

To understand the robustness and limitation of the noise model, simulated data sets with different parameters compositions were generated by using R and then proceeded to identify the significantly VEGs following the flow chart in Fig. 1. Simulation modules implemented were: 1) Master cell gene expression generation; 2) homogeneous cell population gene expression generation (with model parameter *α* and *β*); 3) heterogeneous cell population generation (with model parameter *prct* for number of genes deviated from homogeneous cell population, and *s* for gene expression variation, Eq. 8).

**Fig. 2:**
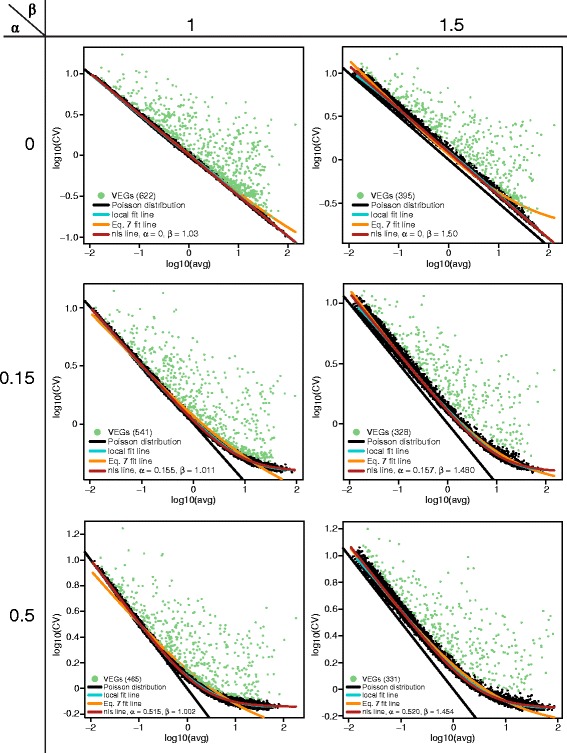
CV-mean plot of data under different *α* and *β*. Other parameters were fixed as gene number = 15,000, cell number = 1,000 cells, *prct* = 10 %, and *s* = 2

The VEG analysis algorithm will first estimate model parameter *α* and *β* described in Eq. 4 by using a cascade of regression (local fit, subsampling, and nonlinear least-squares). For single-cell gene expression data, in the ideal condition all genes should obey CV = *μ*^− 1/2^ [11], following a Poisson distribution as depicted by a black diagonal line in log(*μ*) vs log(CV) plot shown in Fig. 2. In reality, the variance typically exceeds the sample mean, justifying the negative binomial distribution in many NGS applications (and in our simulation example, Eq. 5. The cyan curve in Fig. 2 is the likelihood model of robust local regression using the function locfit.robust in R where outliers were iteratively identified and down-weighted, which allowed us to accurately fit a baseline for the data. The red line in Fig. 2 is the fitted homogeneous variation model and the orange line is the noise model in Eq. 9. With the estimated model parameters $\hat{\alpha}\ \mathrm{and}\ \hat{\beta},$ we will evaluate the regression robustness using RMSE. The parameter estimation process was evaluated by varying initial model parameters (*α* and *β* in Table 1, *s* and *prct* in Table 2, and then number of cells in Table 3) that deviated from master cell population.

**Table 1:** Estimation of model parameters $\hat{\alpha}\  and\ \hat{\beta}$ under different *α* and *β* with fixed number of cells, *prct*, *s*, and gene number = 15,000. Comparing with the noise model in Eq. 9, we have obtained fairly low RMSE in each condition

Simulation parameters					Regression results			
# of cells	*Prct* (%)	*s*	*α*	*β*	$\hat{\alpha}$	$\hat{\beta}$	RMSE	RMSE (Eq. 9)
1,000	10	2	0	1	0.0003 ± 0.0002	1.0293 ± 0.0014	0.0074 ± 0.0006	0.044 ± 0.010
				1.2	0.0004 ± 0.0003	1.2187 ± 0.0024	0.0047 ± 0.0006	0.066 ± 0.020
				1.5	0.0007 ± 0.0004	1.5032 ± 0.0039	0.0028 ± 0.0007	0.091 ± 0.019
			0.15	1	0.1557 ± 0.0005	1.0116 ± 0.0009	0.0047 ± 0.0003	0.049 ± 0.008
				1.2	0.1562 ± 0.0007	1.1965 ± 0.0020	0.0038 ± 0.0004	0.030 ± 0.004
				1.5	0.1569 ± 0.0007	1.4756 ± 0.0047	0.0043 ± 0.0006	0.017 ± 0.001
			0.5	1	0.5146 ± 0.0013	1.0020 ± 0.0010	0.0038 ± 0.0004	0.060 ± 0.006
				1.2	0.5161 ± 0.0011	1.1837 ± 0.0023	0.0039 ± 0.0003	0.047 ± 0.006
				1.5	0.5187 ± 0.0016	1.4561 ± 0.0045	0.0054 ± 0.0005	0.030 ± 0.004

**Table 2:** Estimation of model parameters $\hat{\alpha}\  and\ \hat{\beta}$ under different *prct* and *s* with fixed number of cells, *α*, *β*, and gene number = 15,000

Simulation parameters					Regression results			
# of cells	*α*	*β*	*s*	*Prct* (%)	$\hat{\alpha}$	$\hat{\beta}$	RMSE	RMSE (Eq. 9)
1,000	0.15	1.2	1	10	0.1563 ± 0.0005	1.1965 ± 0.0018	0.0037 ± 0.0003	0.028 ± 0.002
				30	0.1579 ± 0.0006	1.2017 ± 0.0019	0.0048 ± 0.0003	0.026 ± 0.001
				50	0.1612 ± 0.0009	1.2076 ± 0.0023	0.0071 ± 0.0005	0.027 ± 0.001
			2	10	0.1563 ± 0.0005	1.1961 ± 0.0019	0.0040 ± 0.0005	0.033 ± 0.007
				30	0.1612 ± 0.0017	1.2015 ± 0.0024	0.0077 ± 0.0014	0.036 ± 0.001
				50	0.1713 ± 0.0014	1.2080 ± 0.0024	0.0147 ± 0.0012	0.050 ± 0.002
			3	10	0.1572 ± 0.0012	1.1963 ± 0.0026	0.0056 ± 0.0009	0.048 ± 0.008
				30	0.1649 ± 0.0010	1.1997 ± 0.0027	0.0122 ± 0.0011	0.054 ± 0.002
				50	0.1775 ± 0.0012	1.2078 ± 0.0030	0.0225 ± 0.0011	0.096 ± 0.003

**Table 3:** Estimation of model parameters $\hat{\alpha}\  and\ \hat{\beta}$ under number of cells with fixed *α*, *β*, *prct*, *s*, and gene number = 15,000

Simulation parameters					Regression results			
*Prct* (%)	*s*	*α*	*β*	# of cells	$\hat{\alpha}$	$\hat{\beta}$	RMSE	RMSE (Eq. 9)
10	2	0.15	1.2	50	0.1595 ± 0.0024	1.1078 ± 0.0040	0.0127 ± 0.0007	0.037 ± 0.007
				100	0.1587 ± 0.0023	1.1416 ± 0.0044	0.0085 ± 0.0009	0.034 ± 0.006
				500	0.1575 ± 0.0009	1.1836 ± 0.0036	0.0047 ± 0.0008	0.032 ± 0.008

### Estimation of model parameters (*α* and *β*)

We firstly fixed the data set size 15,000 genes and 1,000 cells with *prct* = 10 % and *s* = 2, only the model parameters *α* and *β* were changed, and the fit results of simulation data are shown in Fig. 2. When *α* = 0 and *β* = 1, we simply simulated the data in a Poisson distribution, following a diagonal line in the figure. When *α* became larger, the curve angled more prominent, which indicated data deviated from Poisson distribution at the larger expression level. The increase of *β* resulted in the entire data shifting away from the diagonal line, which might be associated with different sources of technical noises. We observed the robust parameter estimation as shown in Table 1 in all initial model parameters (with RMSE less than 0.01 for all these simulated cases). We noted that sometimes the current model failed to fit a straight line when *α* = 0, which we will investigate further for regression procedures at higher expression level specifically.

When the input parameter *β* became larger, the two estimated model parameters were deviated from the input parameters. However, even in the extreme case where *α* = 0.5 and *β* = 1.5, the RMSE still very consistent in our model (0.0054 ± 0.0005, see Table 1). On the other hand, the orange line - the simple noise model fitting using Eq. 9, can hardly fit the baseline of the simulated data, which results in high RMSE (~0.05, 10x larger than our proposed method) in most conditions.

We further examined the number of significant VEGs under each condition. The pale green points in the log(*μ*)-log(CV) plots in Fig. 2 were the selected as significant VEGs with FDR < 0.05. In the ideal condition where *α* = 0 and *β* = 1, there are in average 940 genes changed by at least two fold change and we have detected around 700 VEGs. Along with the increase *α* and *β*, the number of significant VEGs decreased. In the condition where *α* = 0.5 and *β* = 1.5, there are only around 250 VEGs detected, where around 950 genes are altered by at least two fold change. It is reasonable since the data are more disperse when *α* and *β* become larger. The dispersion affects the fitted normal distribution of CV difference while determining the *p*-value for VEGs, which results in worse FDR when the model parameters are large.

### Test estimation robustness with varying degree of heterogeneity of cell population

Next we tested the performance of model under different percentage of genes affected by random log2 fold change values, which were generated by a normal distribution with zero mean and standard deviation *s* (Eq. 8). The data set size was still set as 15,000 genes and 1,000 cells, and we fixed the model parameters where *α* = 0.15 and *β* = 1.2. From the results in Table 2, we could observe that model parameter *β* is mostly identical and remained close to 1.2 under different levels and numbers of variable genes. However, the model parameter *α* became larger (from 0.156 to 0.178) with the increments of *s* and *prct*. This is unavoidable because *α* represents the dispersion of the data set. With more genes deviated from the homogeneous population, the dispersion increased and estimated *α* biased from the input model parameter value. Due to the deviation of *α*, RMSE also increased when *s* and *prct* became larger. We concluded that the scale and number of variable genes influence the estimation of model parameter *α*, which results in the increase of RMSE. Nevertheless, this issue is solved during the determination of the distribution of CV difference, where we use kernel density to adjust the center of the normal distribution.

### Test estimation robustness with varying number of cells

At last, we would like to know if the model could be properly fit with limited number of cells. We reduced the population size to 50, 100, or 500 cells. To test under a moderate variation condition, we set *prct* = 10 %, and *s* = 2, with model parameters remained as *α* = 0.15 and *β* = 1.2. The results in Table 3 show that reducing the number of cells slightly affected the estimation of *α*: *α* is larger when the number of cells is smaller, in which CV of genes are more disperse. The estimation of *β* also deviated a bit with the decrease of the population size. Under 50 and 100 cells conditions, the scattering of the data points around the diagonal line resulted in the estimation error of *β* and a higher RMSE in lower number of cells. Moreover, the two factors that influenced the estimation of *α* and *β* also played a role in calling significant VEGs. Under the same number of genes, we determined only about 355 VEGs in 500 cells condition, whereas about 596 VEGs were called in 50 cells condition. With only a small number of cells, the normal distribution of homogeneous genes is difficult to estimate accurately, which might result in the increase or decrease of detected VEGs. Hence, a sufficient number of cells is necessary to accurately determine VEGs among a cell population.

In conclusion, the major factors that influence the robustness of the noise model are how data distributes and the number of cells. Fitting errors arise from two situations, 1) the estimated parameters are unusually large (especially *β*) in the simulated cases, which is unlikely in real scRNA-seq data, 2) the data distribute close to the diagonal line in the CV-mean plot, but with many variable genes at higher expression level, which results in the failure of fitting a straight line. The cell population size is also a concern; however, in reality a single-cell experiment should be designed with a large number of cells. Hence, the population size may not be a major factor for most single cell applications.

From the simulation results we could find out that a simple fitting method is not enough. By fitting the model in Eq. 9 straightforwardly, we got much larger RMSE in every condition. In contrast, our expression variation model design with multiple layers of estimates can be fitted properly for most of the experiment condition. However, in some cases the fitted model curve (red) lay under the local fit curve (cyan) at the middle mean abundance interval, which it might be a potential problem occasionally.

### Application on real data sets

**Fig. 3:**
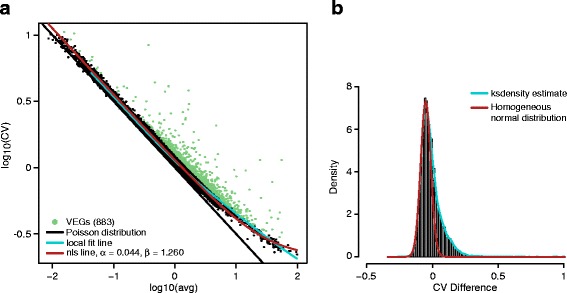
**a** CV-mean plot of data set GSE65525 and **b** the CV difference histogram

We have identified the VEGs for the two scRNA-seq data sets, and the respective CV-mean plots are shown in Fig. 3. From Fig. 3a, we can see that most genes in the first data set (GSE65525) distribute nearby the diagonal line, inferring that the data were only affected slightly by technical noises. Part of the fitted model overlaps with the Poisson distribution line until the mean abundance is larger than 1. Foreseeably, the two model parameters are close to the ideal case, we estimated that *α* = 0.044 and *β* = 1.260. In Fig. 3b, the cyan line is the kernel density estimation of CV difference to find the peak of the normal distribution of homogeneous genes. Using the left side of the peak, the red line is the fitted normal distribution and we identified 883 VEGs with FDR less than 0.001.

**Fig. 4:**
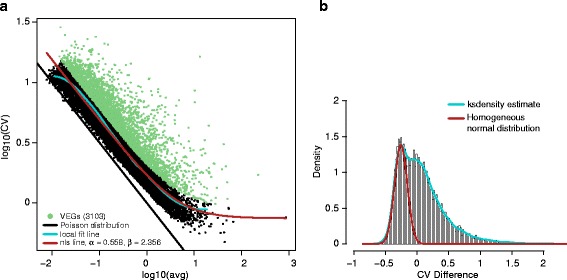
**a** CV-mean plot of data set GSE60361 and **b** the CV difference histogram

The second data set, GSE60361 shown in Fig. 4a, is much more disperse and deviated away from the diagonal line. However, our method still fitted a reasonable noise model. Even though the local fit curve was terminated around *μ* = 10, the extension of the noise model at tail interval fitted well. The model parameters where *α* = 0.558 and *β* = 2.356 are much larger, and the histogram of CV difference is also widely distributed. Similar with the simulation case with high percentage of variable genes, the fitted model can't locate accurately on the center of the normal distribution of homogeneous genes. In Fig. 4b, we estimated the normal distribution where the peak is around −0.2. As a result, 3103 genes were defined as VEGs, which is a very large number. We found out that the average UMIs of each cell in the second data set is only around 14,000, which is far less than the first data set with around 29,500 UMIs. The small number of UMI counts results in large dispersion of data and detecting a large number of VEGs. Clearly, the total UMI reads per cell in this data is too small to obtain a precise estimation of model parameters. Additional simulation perhaps is needed to further evaluate the requirement of effect of number of UMIs for single cell study.

### Determination of single-cell subpopulations

**Fig. 5:**
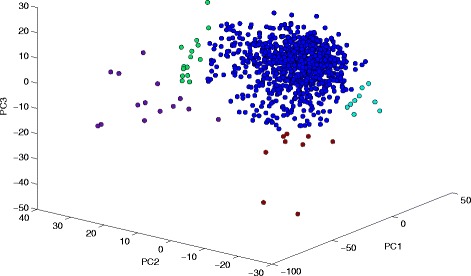
3-D PCA plot of data set GSE65525

After the determination of VEGs, we can use different conventional bioinformatics tools to further study the heterogeneity and subpopulation of single-cell population. Principal component analysis (PCA) can be used to find out possible subpopulations among the entire single-cell population. Here we picked the first data set to demonstrate the subsequent scRNA-seq analysis. First, we used principal component analysis (PCA) on the log-transformed data of 883 selected genes to observe the heterogeneity among all cells, shown in Fig. 5. We could find some possible subpopulations at the left, top left, right, and bottom corners, which were labeled in different colors in Fig. 5 After we determined subpopulations from the PCA result, other methods can be applied to study the heterogeneity of the cell population: using the principal component (PC) loadings to classify the genes; or using Single-Cell Differential Expression (SCDE) [19] and/or DESeq [20] algorithms to identify differential expressed (DE) genes between different subpopulations. We can further perform functional annotation and pathway analyses on identified DE genes to understand the origins of cell heterogeneity.

Even though the two scRNA-seq experiments obtained from GEO database used two different techniques to capture single cells with vastly different distributions in the CV-mean plots as shown in Figs. 3 and 4 , we could fit the expression variation models properly for both data. In the previous two studies [11, 12], it has been demonstrated that, using VEGs, cell heterogeneity has been detected along with associated biological functions of subpopulations. Clearly, finding the VEGs of a single-cell experiment is just the first step. The subsequent analyses that utilizing VEGs and their expression changes across the cell population are the key of single-cell RNA-seq analysis.

## Conclusion

In this paper, we proposed a single cell gene expression variation model, and demonstrated the method to regress the model parameters for a single-cell RNA-seq experiment by exploiting the relationship between the coefficient of variation and mean transcript abundance of all genes in the genome. A single-cell data simulation was also designed and used to determine the robustness of the model parameter estimation. In most condition the model parameters were estimated precisely, and resistant to the influence of factors such as population size, and dispersion of genes. The results of testing on two real scRNA-seq data sets further confirmed our simulation, while additional modeling requirement due to lower total UMI count per cell warrants further investigation.

## Abbreviations

CV, Coefficient of Variation; DE, Differential Expression; DEG, Differentially Expressed Gene; GEVM, Gene Expression Variation Model; NB, Negative Binomial (distribution, model); NGS, Next Generation Sequencing; PC, Principal Component; PCA, Principal Component Analysis; RMSE, Root-mean Square Error; scRNA-seq, single-cell RNA-seq; UMI, Unique Molecular Identifier; VEGs, Variably Expressed Genes
